# Gut microbiota profiling revealed the regulating effects of salidroside on iron metabolism in diabetic mice

**DOI:** 10.3389/fendo.2022.1014577

**Published:** 2022-09-23

**Authors:** Jing Shi, Qin Zhao, Dou Dou Hao, Hong Xia Miao, Sha Wan, Chao Hua Zhou, Si Yu Wang, Si Yuan Chen, Jin Shang, Tian Hang Feng

**Affiliations:** ^1^ Hospital of Chengdu Office of People’s Government of Tibetan Autonomous Region (Hospital.C.T.), Chengdu, China; ^2^ Department of Laboratory Medicine, Qingdao Central Hospital, Qingdao, China; ^3^ Sichuan Provincial People’s Hospital, University of Electronic Science and Technology of China, Chengdu, China

**Keywords:** salidroside, diabetes, gut microbiota, iron metabolism, lactobacillus

## Abstract

**Background:**

Diabetes is a common metabolic disease that is associated with gut microbiota dysbiosis and iron metabolism. Salidroside (SAL) is the main ingredient of the traditional Chinese herb Rhodiola, previous studies have shown that SAL could reshape the gut microbiota and limit iron accumulation. Therefore, it is possible that SAL can act as an alternative therapy for diabetes, and its underlying mechanism is worth exploring.

**Methods:**

SAL was used to treat diabetic db/db mice. Serum glucose and iron levels and the histopathology of myocardial fibres were evaluated. The gut microbiota composition was determined by 16S rRNA Illumina sequencing technology.

**Results:**

Treatment with SAL significantly reduced blood glucose and ameliorated diabetic cardiomyopathy in diabetic db/db mice, which was accompanied by inhibited ferroptosis and iron accumulation. Furthermore, the 16S rRNA sequencing results showed that SAL induced a change in the gut microbiota composition. Overall, SAL could increase the proportion of probiotic bacteria and decrease Lactobacillus to improve gut microbiota. Specifically, SAL increased the ratio of Bacteroidetes to Firmicutes in diabetic mice. The most significant biomarker was the genus Lactobacillus between the MD group and the SAL group. In addition, COG and KEGG analyses suggested that SAL mainly participated in nutrient metabolism, among them iron metabolism was associated with the abundance of Lactobacillus.

**Conclusions:**

SAL could reduce the glucose level and protect against diabetic cardiomyopathy in diabetic mice, which might be mediated by the change in the gut microbiota and the regulation of iron metabolism. The findings suggested that SAL was a promising complementary option for diabetes therapy.

## Introduction

Diabetes mellitus (DM) is a chronic disease with a high disability rate, and DM is the ninth leading cause of death. The International Diabetes Federation estimated that 1 in 11 adults aged 20–79 years (463 million adults) had DM worldwide in 2019 and that the number would increase to 578 million by 2030 and, surprisingly, 700 million by 2045. Moreover, according to previous reports, the actual prevalence of DM is often underestimated, and the global prevalence of DM might be even grimmer (1). Type 2 diabetes mellitus (T2DM) accounts for the vast majority (approximately 90%) of DM cases. T2DM is characterized by a combination of relative insulin deficiency and insulin resistance. In terms of pathophysiological manifestations, blood glucose is elevated, and the metabolism of a range of nutrients can also be affected (2). Among the risk factors for T2DM, such as genetic factors and high caloric intake, the role of the gut microbiota in the progression of T2DM has been gradually revealed (3).

The gut microbiota is recognized as a key factor in human health. Previous studies have shown that many chronic diseases might lead to gut microbiota disorders (4). A healthy gut microbiota is beneficial for the host, but gut microbiota changes are associated with a range of metabolic dysbioses, including diabetes and obesity (5). Gut microbiota dysbiosis in diabetes patients is linked to inflammation status, oxidative stress, and toxin release. Hence, probiotics might represent a novel, economical approach for managing T2DM (6). In addition, the gut microbiota is associated with other factors involved in T2DM, such as nutrient metabolism.

Iron is an important nutrient with extensive physiological functions for both the host and the gut microbiota (7). Iron overload can lead to the formation of toxic oxygen free radicals (hydroxyl radicals) through the Fenton reaction. Thus, iron metabolism needs to be tightly regulated at the cellular and organism levels (8). Abnormal iron metabolism in T2DM has been observed in a large number of cross-sectional surveys and further verified by emerging cohort studies (9). Iron metabolism has been reported to play an important role in reshaping the gut microbiota in T2DM (10). Therefore, the regulation of both the gut microbiota composition and iron metabolism is essential in ameliorating insulin resistance and improving the prognosis of T2DM.

Rhodiola is a kind of traditional Chinese herb that is recorded in the Ming Dynasty classics Compendium of Materia Medica. It is mainly grown at altitudes of 1600~4000 metres and is commonly distributed in Chinese Tibet (11). Rhodiola rosea has a long history of use as an adaptogen in traditional Chinese medicine and is also called “Tibetan ginseng”. The function and application prospect of Rhodiola as a Chinese herb were first summarized in 2007 (12). Salidroside (SAL) is one of the most potent bioactive constituents isolated from Rhodiola. Recent studies have shown that SAL has many attractive physiological functions, including reducing the risk of T2DM (13). Regarding the mechanism by which SAL reduces the risk of T2DM, SAL was reported to significantly reduce the level of oxygen free radicals by inhibiting the oxidative stress response; for example, SAL showed a protective effect in myocardial cells, with changes in a range of antioxidant enzymes. The antioxidant enzymes were also reported to be involved in iron metabolism (14). On the other hand, SAL exhibited functions in regulating the living environment of the gut microbiota so that the proportion of beneficial bacteria was increase (15). Therefore, SAL potentially has an effect on the regulation of both the gut microbiota and iron metabolism. The role and mechanism of SAL in DM is worthy of exploration. Here, we designed the present study to explore the effect and concrete mechanism of SAL on the gut microbiota and iron metabolism in T2DM, aiming to provide new ideas for the prevention and treatment of T2DM.

## Materials and methods

### Chemicals and reagents

Salidroside was purchased from Chengdu Refines Biotechnology Co., Ltd. (Chengdu, China). Anti-GPX4 rabbit monoclonal antibody (ab125066), anti-SCL7A11 rabbit monoclonal antibody (ab175186) and anti-cTNT rabbit monoclonal antibody (ab209813) were purchased from Abcam (Cambridge, UK), and anti-GAPDH rat monoclonal antibody was purchased from Affinity (USA).

### Animal experiments

Twenty male BKS-Leprem2Cd479/Gpt (db/db)(C57bl/KS diabetic mice with knockdown of the Leptin receptor gene) and ten male BKS-Leprem2Cd479/Gpt (wt/wt) (C57bl/KS wild-type normal mice), the standard was 8 weeks of age, were purchased from Jiangsu Jicui Yaokang Biotechnology Co., Ltd. (Jiangsu, China). The db/db mice with blood glucose levels that were over 16.7 mmol/L, as measured by an Accu-Chek Performa glucometer (Roche Diagnostics, Mannheim, Germany), for 3 days were considered diabetic mice. The experiment was started after a 1-week period of acclimation to laboratory conditions. Ten wt/wt mice were normal controls (the WT group), and twenty db/db mice were randomized into 2 groups, namely, (1) the SAL group and (2) the T2DM model group (MD group), with ten mice in each group. The SAL group was intragastrically administered SAL (1.5 g/kg) for 5 weeks, whereas the MD and WT groups were both administered the same amount of normal saline. The dosage of SAL was determined according to a previous SAL toxicity study (16). The animals of each group were provided with their corresponding diet and were allowed free access to tap water. Body weight and blood glucose were measured weekly. At the end of the experiments, animals were intraperitoneally injected with 6% chloral hydrate (0.5 mL/10 g), and blood was taken from the veins. Serum was separated and then stored at −80°C for biochemical estimations. The heart was dissected and stored at −80°C for further analysis, and the faeces of mice were stored at −80°C for intestinal microflora analysis. All experiments performed were approved by the ethics committee of the Hospital of the Chengdu Office of the People’s Government of the Tibetan Autonomous Region.

### Sequencing of the gut microbiota

Faeces were collected from animals before they were dissected. Then,DNA extraction kit was purified by the Zymo Research BioMICS DNA Microprep Kit(Cat# D4301), and the DNA concentration was quantified. The 16S rRNA V4 region of the sample was amplified, detected, purified and quantified. Then, New England BioLabs NEBNext Ultra II DNA Library Prep Kit for Illumina (NEB#E7645L) and a HiSeq Rapid SBS Kit v2 (FC-402-4023 500 Cycle) and Illumina sequencing technology were used for sequencing (17). Finally, the data obtained by sequencing were used for bioinformatics analysis using Usearch and QIIME1.9.0 (18).

### Determination of the biochemical parameters

Serum levels of iron, ferritin and transferrin were detected by the Hospital of the Chengdu Office of the People’s Government of the Tibetan Autonomous Region using the corresponding colorimetric kit according to the manufacturer’s instructions.

### Pathology analysis

Heart tissues were fixed in 4% paraformaldehyde for 48 h. Fixed tissues were processed for dehydration and paraffin embedding, and 3-µm-thick sections were prepared for histopathological and immunohistochemical examinations. Heart sections were stained with haematoxylin and eosin (H&E) for routine histopathological examination as well as morphometric analysis with a light microscope (Nikon, Japan) at 200× magnification and a panoramic slice scanner (3DHISTECH, Hungary). The specimens were placed under a high-power optical microscope, and 20 fields were randomly selected. Then, the mean value of the grading of myocardial injury was taken based on the following scale: Grade 0 – the muscle fibres were neatly arranged, the transverse lines were clear, the nuclei were obvious, and there was no cell swelling; Grade I – scattered point-like necrosis of the myocardium, mainly coagulative necrosis, confined to the subendocardial; Grade II – the myocardial necrosis foci were distributed in sheets without connections, and the lesions did not involve the whole layer of the heart wall; and Grade III – extensive patchy necrotic of the myocardium, interconnecting with the heart and involving the whole pericardium. Immunohistochemical examination was used to detect the GPX4 and cTNT content and distribution. The sections were observed at 200× magnification. Then, the optical density value (IOD) and the pixel area (AREA) of the brown-coloured immunostained area were measured.

### Western blot analysis

The heart tissue was washed 2-3 times with cold PBS to remove blood, was cut into small pieces and placed in a homogenizer. Then, RIPA lysis solution at 10 times the tissue weight was added, and the tissue was homogenized thoroughly on ice and centrifuged at 12,000 × g for 10 min. The supernatant was collected to form the total protein solution. The protein concentrations were measured by a BCA protein assay kit (Biosharp, Shanghai, China). Frozen protein sample was removed and thawed on ice, and a 4× volume of the total protein was loaded onto a 10% SDS acrylamide gel and then electrotransferred to a polyvinylidene fluoride (PVDF) membrane (Millipore, ISEQ00010, USA). After blocking with 5% skim milk (TBST preparation) for 1 h at room temperature, the membranes were incubated overnight at 4°C with the primary antibody against the target protein and then for 1 h with the corresponding secondary antibody. The PVDF-membrane-bound protein was placed on the sample tray and detected with an ECL Plus kit according to the operating guidelines. Then, images were collected under a chemiluminescence imaging system (ChemiScope 6100, Shang Hai, China), and the density of each target band was quantified by Image Pro-Plus 6.0 software (Media Cybernetics, Inc., Rockville, MD, USA), with the optical density normalized to that of GAPDH.

### Transmission electron microscopy

The heart tissue of the animal was fixed in glutaraldehyde (3%) and then fixed again in 1% osmium tetroxide. Subsequently, the samples were stepwise dehydrated, permeabilized, and embedded in Araldite. Then, the tissues were cut into ultrathin sections (50 nm) using an ultramicrotome (Leica EM UC7, Solms, Germany) and placed onto a copper grid. Subsequently, the thin slices on the copper grid were stained with uranyl acetate (2%) and lead citrate at room temperature for 15-20 min and observed under a transmission electron microscope (JEM-1400FLASH, JEOL, Japan).

### Statistical analysis

All animal data are expressed as the mean ± standard deviation (SD). For between-group comparisons in this study, we used one-way analysis of variance (ANOVA) followed by Tukey’s *post hoc* test. All data were analysed using the statistical program SPSS version 21 (SPSS Inc., Chicago, IL, USA). Graphs were created using GraphPad Prism version 8 (San Diego, CA, USA). Differences with a P value < 0.05 were considered statistically significant differences.

## Results

### SAL reduced the levels of blood glucose and protected against diabetic cardiomyopathy in db/db mice

**Figure 1:**
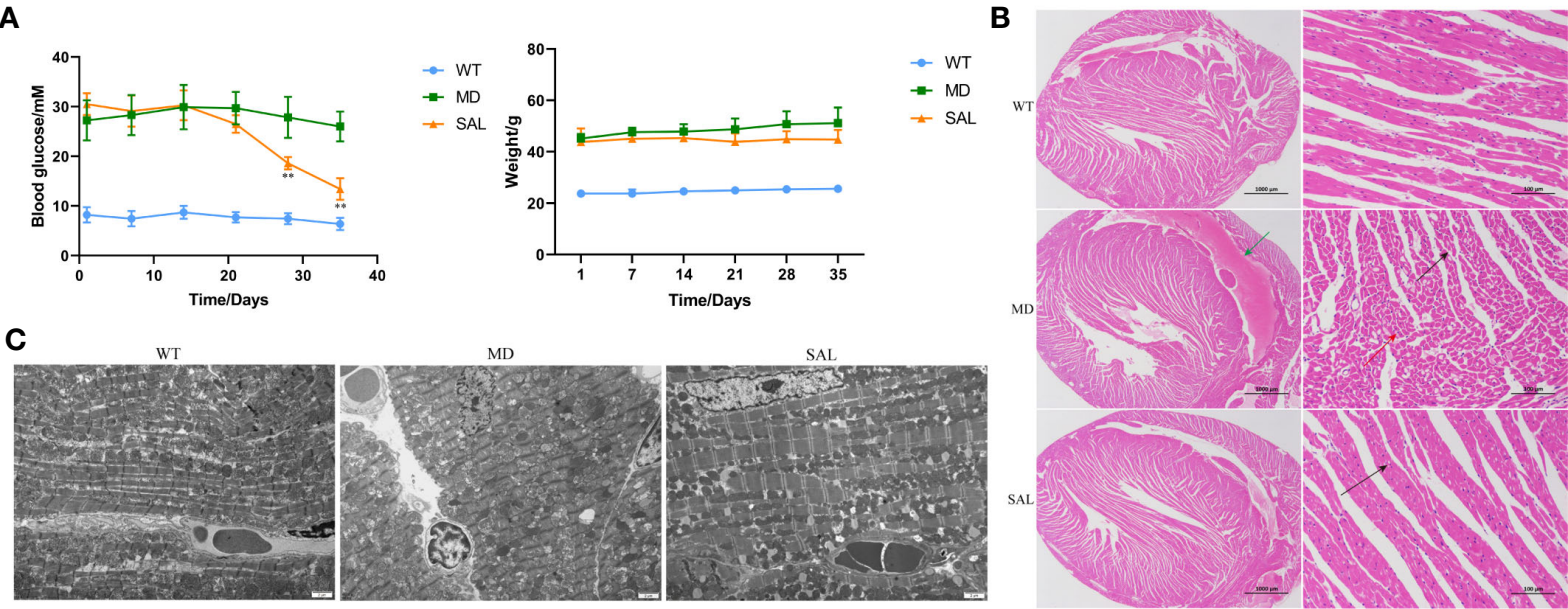
Influence of SAL on glucose regulation and diabetic cardiomyopathy in diabetic mice. **(A)**, Changes in the blood glucose and weight of db/db mice during the period of SAL treatment. (**p < 0.05 as MD group vs SAL group). **(B)**, H&E staining of myocardial tissue sections. **(C)**, Transmission electron microscopy of myocardial cells.Green arrow: extravasation of the heart cavity; black arrow: irregularly shaped cavities; red arrow: necrotic cardiomyocytes.

Ten wt/wt mice formed the WT group, and twenty db/db mice were randomized into 2 groups, namely, (1) the SAL group and (2) the T2DM model group (MD), with 10 mice in each group. After 35 days, there were two mice that died in the MD group and three mice that died in the SAL group. SAL did not exhibit toxicity to mice in the SAL group compared with the control group. The levels of baseline glucose in db/db mice in the MD group and the SAL group were similar and significantly higher than those in wt/wt mice in the WT group. The treatment of db/db mice with SAL reduced their blood glucose during the intervention period. A significant reduction in blood glucose was observed in the SAL group after week 4 when compared with that in the MD group (**Figure 1A**). The weight of db/db mice in the SAL group was slightly lower than that of the MD group, but the difference was not significant, suggesting that treatment with SAL tended to reduce the weight of db/db mice (**Figure 1A**). These results showed that SAL could alleviate elevated blood glucose in diabetic mice.

The histopathology of myocardial fibres was observed, and the heart tissues of each mouse were used to make paraffin sections. The H&E staining results showed that no pathological changes existed in normal mice in the WT group. The myocardial cell boundaries were clear, the shape was consistent, and the grade of myocardial injury was Garde 0. Uniform and unstructured coagulation of necrotic myofibrils was found in db/db mice in the MD group (**Figure 1B**, green arrow). A number of irregular myocardial fibres and obscure intercellular borders were also found in db/db mice in the MD group (**Figure 1B**, black arrow); the myocardial cell nucleus was hyperchromatic, fragmented or dissolved, and the cytoplasm was eosinophilic (**Figure 1B**, red arrow). The grade of myocardial injury was Grade I. In contrast, SAL ameliorated the myocardial necrosis of diabetic myocardial tissue in db/db mice. Necrotic myofibrils and obscure intercellular borders were rarely found in db/db mice in the SAL group, and the grade of myocardial injury was Grade 0.

To further observe the effect of SAL on myocardial cells in diabetic mice, 6 mice in each group were randomly selected to observe the changes in myocardial cell microstructure by TEM. The results showed that the morphology and structure of cardiomyocytes in the WT group were normal, the myofibrils were arranged neatly, and the M and Z lines of the dark band were clear and straight. In contrast, in the MD group, most mitochondria between myocardial fibres were swollen, the cristae were broken and dissolved, myofibrillary dissolution existed in some regions, the Z line of the dark zone was clear and straight, and there were many lipid droplets and a few secondary lysosomes. In the SAL group, the myofibrils of db/db mice were arranged neatly, the sarcomere structure was intact, and the mitochondria between the myofibrils were normal in shape without obvious lesions (**Figure 1C**). A characteristic of ferroptosis is mitochondrial dysfunction, including mitochondrial swelling and cristae dissolution. The results indicated that the alleviation effect of SAL on mitochondrial injury was associated with ferroptosis.

### SAL inhibited ferroptosis by reducing iron overload in db/db mice

**Figure 2:**
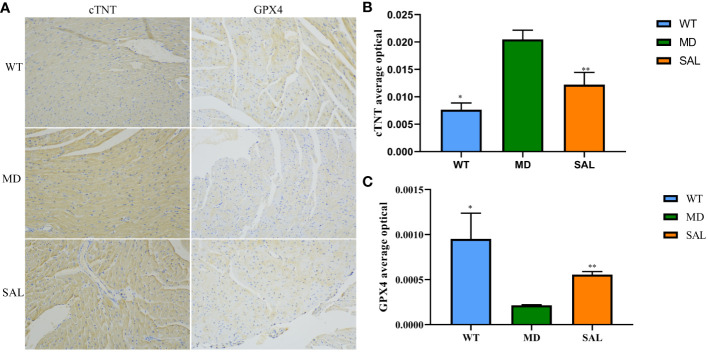
Influence of SAL on the expression of myocardial injury markers in diabetic mice. **(A)**, Immunohistochemistry of cTNT and GPX4 in myocardial tissue. **(B)**, The optical density value (IOD) of the brown colour of cTNT staining in myocardial tissue. **(C)**, The IOD value of the brown colour of GPX4 staining in myocardial tissue. (ns, no statistical significance; *p < 0.05 as MD group vs WT group; **p < 0.05 as MD group vs SAL group).

To further investigate the influence of SAL on ferroptosis in diabetic mice, immunohistochemistry analysis of cTNT and GPX4 was conducted (**Figure 2A**). The expression of cTNT was higher in the MD group than in the WT group (*p*=0.000) and SAL group (*p*=0.001) (**Figure 2B**). This finding indicated that diabetic myocardial damage was present in the diabetic mice. The expression of GPX4 was lower in the MD group than in the WT group (*p*=0.002) and SAL group(*p*=0.045) (**Figure 2C**). GPX4 is an inhibitor protein of ferroptosis. This finding indicated that SAL could inhibit ferroptosis.

**Figure 3:**
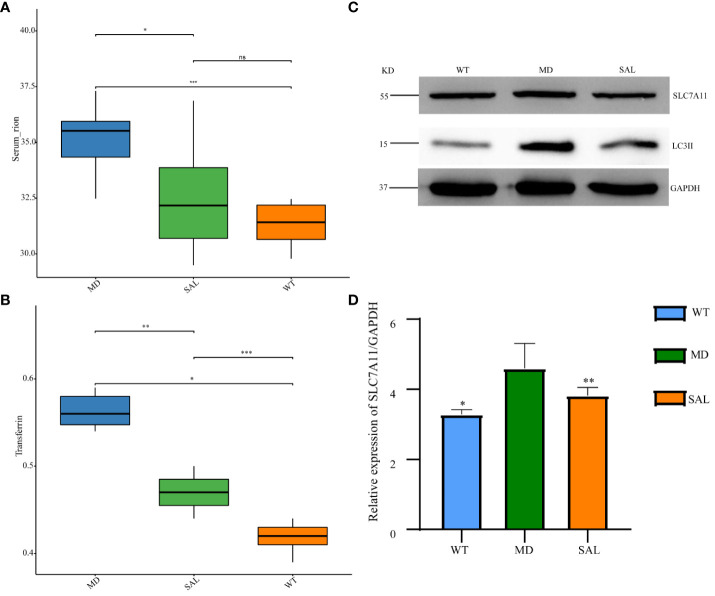
Influence of SAL on iron metabolism in diabetic mice. **(A)**, The serum iron levels of mice in the different groups; **(B)**, The transferrin levels of mice in the different groups; **(C)** Western blot analysis of the protein expression of SLC7A11, LC3II, and GAPDH in myocardial tissue; **(D)** Grey value analysis of the western blot results. (ns, no statistical significance; *p < 0.05 as MD group vs WT group; **p < 0.05 as MD group vs SAL group). ***means that SAL group compared with WT group is difference significant.

The occurrence of ferroptosis depends on iron overload and oxidative stress injury. To further explore the effect of SAL on iron metabolism, the levels of serum iron and transferrin were detected. The results showed that transferrin and serum iron were higher in the MD group than in the WT group. The levels of serum iron and transferrin were significantly lower in the SAL group than in the MD group (**Figure 3A, B**). This finding indicated that SAL could reduce iron overload in diabetic mice. Western blotting of two protein markers of iron metabolism, SLC7A11 and LC3II, was conducted. The results showed that the expression of LC3II was higher in the MD group than in the WT group. SAL reduced the expression of LC3II in diabetic mice (**Figure 3C**). Moreover, SAL tended to reduce SLC7A11 expression *via* grey value analysis (**Figure 3D**).

### SAL exerted a probiotic effect in the regulation of the gut microbiota in db/db mice

**Figure 4:**
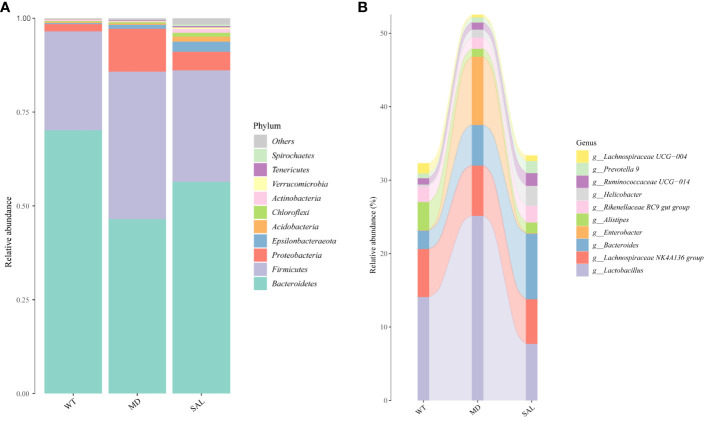
Changes in the gut microbiota of mice in the different groups. **(A)**, The overall composition of the gut microbiota at the phylum level. **(B)**, Sankey plot of the changes in the composition of the gut microbiota at the genus level.

To analyse the effect of SAL on the gut microbiota of diabetic mice, 16S rRNA sequencing was conducted. The sequencing data have been published in NCBI (https://www.ncbi.nlm.nih.gov/sra/PRJNA868147) and the code is PRJNA868147. We obtained a total of 756,702 clean tags from 768,415 raw tags (Supplement 1). The results showed that the most abundant phylum in any group was Bacteroidetes, which can benefit hosts by preventing infection with potential pathogens in most instances (19). The proportion of Bacteroidetes was decreased in diabetic mice in the MD group compared with mice in the WT group, while SAL ameliorated the reduction in Bacteroidetes in diabetic mice in the SAL group. The second most abundant phylum was Firmicutes. The proportion of Firmicutes was increased in diabetic mice in the MD group compared with mice in the WT group, while SAL ameliorated the up-regulation of Firmicutes in diabetic mice in the SAL group. The ratio of Bacteroidetes to Firmicutes was decreased in the MD group, while the ratio was increased after SAL treatment in the SAL group. Other abundant phyla included Verrucomicrobia, Proteobacteria, Actinobacteria, Tenericutes, Desferribacteres, Cyanobacteria and several unclassified bacteria (**Figure 4A**). A decrease in Bacteroidetes or an increase in Firmicutes or a decrease in the ratio of Bacteroidetes to Firmicutes contributes to the promotion of obesity and increases the risk of diabetes (20). Therefore, this result showed that the SAL could reduce the risk of diabetes.

At the bacterial genus level, the genera *Enterobacter*, *Lactobacillus,bacteroides, lachnospiraceae NK4A136 group and alistipes accounted for a larger proportion.* Among these, *Enterobacter* and *Lactobacillus* were increased, *bacteroides* and *alistipes* were decreased in the MD group compared with the SAL group, while SAL ameliorated the up-regulation of the genera *Enterobacter* and *Lactobacillus* in diabetic mice in the SAL group (**Figure 4B**). So, we speculated that SAL could up-regulate the proportion of probiotic bacteria (*bacteroides* and *alistipes)* and reduce the proportion of pathogenic bacteria (*Enterobacter)* in diabetic mice, and also has a unique down-regulation effect on *Lactobacillus*.

### *Lactobacillus* was a potential biomarker of SAL treatment

**Figure 5:**
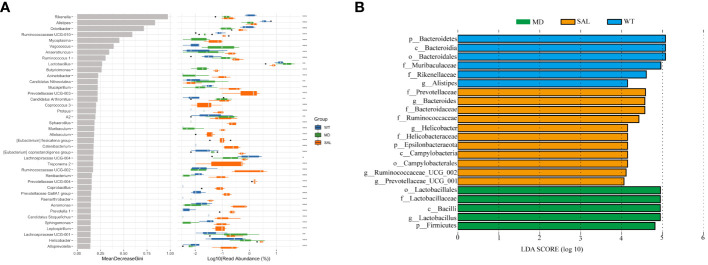
Assessment of significantly different species between groups. **(A)**, Random forest analysis was combined with a difference test indicating significant differences in different groups and the importance of classification between groups. The * on the right represents the significance of the difference between groups (Kruskal–Wallis rank-sum test) (\***p < 0.001; \**p < 0.01; \*p < 0.05). **(B)**, LDA scores plot. The horizontal coordinates were the logarithmic scores of LDA for each classification unit. The vertical coordinates were the classification units with significant difference between groups. The longer the length, the more significant the difference was.

To further explore the key biomarkers associated with SAL treatment, random forest analysis was conducted. The results showed that the proportions of *Lactobacillus* was significantly higher in the MD group than in either the WT group or the SAL group. In contrast, the proportions of *Odoribacter, Ruminococcaceae UCG-010*, and *Lachnospiraceae UCG-004* were significantly lower than those in either the WT group or the SAL group (**Figure 5A**). Moreover, the linear discriminant analysis (LDA) effect size (LEfSe) method was performed. The histogram of the distribution of LDA values (LDA > 2.5) showed the significantly different biomarkers (*p* < 0.05). The results showed that *Lactobacillus* was the most significant difference and could be used as a biomarker of diabetes by comparing the MD group with the SAL (*p*=0.002), which coincidentally indicated that *Lactobacillus* was significantly down-regulated in SAL group after SAL treatment (**Figure 5B**). Therefore, the above results suggested that members of the genus *Lactobacillus* were the unique target bacteria of SAL treatment.

### *Lactobacillus* was associated with iron metabolism 

**Figure 6:**
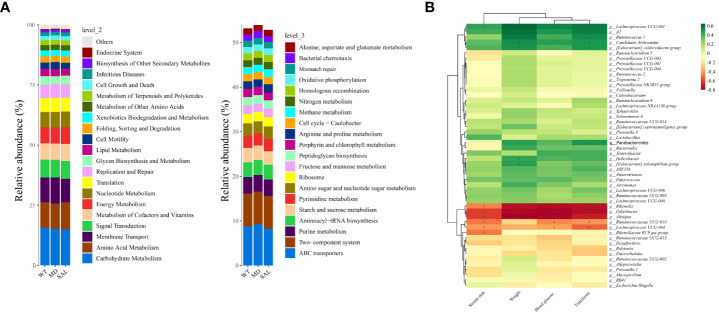
Functional prediction and analysis of SAL-related bacteria. **(A)**, Kyoto Encyclopedia of Genes and Genomes (KEGG) pathways by Tax4Fun tools at level 2 and level 3. **(B)**, Correlation analysis between high-abundance genera and iron metabolism and glucose metabolism. Color depth indicates correlation, the more red indicates the stronger negative correlation, the more green indicates the stronger positive correlation, and the asterisk indicates significance (\**p < 0.01; \*p < 0.05).

To explore iron metabolism-related bacteria, the Cluster of Orthologous Groups of proteins (COG) and the Kyoto Encyclopedia of Genes and Genomes (KEGG) pathway databases were used to make functional predictions. The significant differences in species between each group were analysed by LEfSe. The threshold values of LDA > 2.5 and p < 0.05 were used. The results showed that SAL mainly participated in nutrient metabolism, such as glycan biosynthesis and metabolism and lipid metabolism, at level 2. Furthermore, oxidative phosphorylation, starch and sucrose metabolism, and amino sugar and nucleotide sugar metabolism were involved in SAL treatment at level 3. According to the KEGG database, the significantly different functional pathways were similar to those in the COG categories. Briefly, genes related to nutrient metabolism were enriched in the SAL-treated group (**Figure 6A**).

To further investigate the relationship between the gut microbiota and iron metabolism, correlation analyses between the abundances of different bacterial species and serum iron, transferrin, blood glucose and weight were conducted. The results showed that *Rikenella*, *Alistipes*, *Odoribacter*, etc., *w*ere negatively correlated with serum iron, blood glucose, weight and transferrin. In contrast, *Lachnospiraceae UCG-001, Ruminococcus-1, Candidatus Arthromitus, Enterobacter, Lactobacillus*, etc., were positively correlated with serum iron, blood glucose, weight and transferrin (**Figure 6B**). Among them, only *Lactobacillu*s was down-regulated in diabetic mice after SAL treatment (**Figure 4B**), and also *Lactobacillus* was a biomarker between the MD group and SAL group, and SAL had a down-regulation effect on it (**Figure 5B**). *Lachnospiraceae UCG-001, Ruminococcus-1, Candidatus Arthromitus, Enterobacter* despite showing a positively correlation with iron metabolism, did not show a statistical difference during the comparison between the SAL and MD groups. Among the bacteria commonly related to glucose metabolism and iron metabolism, the abundance of *Lactobacillus* was significantly higher in diabetic mice with serum iron load, which is consistent with the biomarker screening results of SAL treatment. The above results showed that SAL could alleviate iron overload in diabetes by reducing the abundance of *Lactobacillus* in the gut microbiota.

## Discussion

DM involves an array of metabolic dysfunctions due to hyperglycaemia, and these metabolic dysfunctions lead to a series of complications, such as diabetic retinopathy, diabetic nephropathy, diabetic cardiopathy, diabetic coma, and diabetic foot (21). The number of people with DM worldwide continues to increase, which poses an increased risk of all-cause mortality (22). Novel therapeutic strategies that reduce the complications of DM are still worth exploring.

In the present study, we evaluated the function of SAL in treating diabetic mice. *In vivo* tests showed that SAL could reduce the blood glucose level and alleviate diabetic cardiomyopathy in diabetic mice. These results were in accordance with the results of previous studies (22–24). To further investigate the mechanism of SAL, the effects of SAL on ferroptosis occurrence and iron metabolism were evaluated. We found that SAL inhibited the expression of cTNT and GPX4 in the myocardial tissue of diabetic mice. cTNT is an indicator of myocardial injury, while the expression of GPX4 is an inhibitor of ferroptosis and is involved in iron metabolism (25). Furthermore, we found that SAL could limit iron accumulation in diabetic mice and inhibit the expression of SLC7A11 and LC3II. SLC7A11 and LC3II were identified as iron metabolism-related genes (26, 27). This finding indicated that the preventative effect of SAL on diabetic cardiomyopathy was associated with iron metabolism regulation.

Increasing evidence suggests that iron metabolism and the gut microbiota are frequently linked, and dysfunctions of both have been observed in patients with T2DM (28). Moreover, the gut microbiota was reported to act as a regulator of metabolic diseases such as DM. Currently, there are few studies on the relationship between SAL and the gut microbiota in diabetes. Therefore, we explored the gut microbiota composition using 16S rRNA sequencing and evaluated iron metabolism-related intestinal bacterial indicators. In our study, SAL increased *Bacteroides* and reduced *Lactobacillus*. Studies indicated that *Bacteroides* played a beneficial role on glucose metabolism in humans and experimental animals (29).It had also been reported that *Lactobacillus* was increased in T2DM and nonalcoholic fatty liver disease (30). Which indicated that SAL could up-regulate the proportion of probiotic bacteria and down-regulated *Lactobacillus* to improved the gut microbiota. Moreover, at the bacterial genus level, members of the genus *Lactobacillus* were the unique target bacteria of SAL treatment. It is interesting that *Lactobacillus casei* could act as a probiotic in diabetes (31). However, *Lactobacillus* has been reported to increase ferric iron (32). To clarify the association between the gut microbiota and iron metabolism, correlation analysis was conducted between different flora and related indicators of iron metabolism. The results indicated that SAL could alleviate iron overload in diabetes by reducing the abundance of *Lactobacillus*. Therefore, the results of the present study showed that SAL was found to reduce T2DM-associated pathogenic bacteria and that it had a potential effect on preventing diabetic cardiomyopathy by regulating iron metabolism-associated bacteria.

Gut microbiota-targeted therapy is a promising new therapeutic strategy in T2DM patients. Oral administration of probiotics, prebiotics or faecal microbiota could improve gut metabolic functions during the treatment of T2DM. However, the use of antibiotics restricts their efficacy (33). On the other hand, no-drug therapy, including diet modification and exercise, could also benefit T2DM patients by influencing the gut microbiota. For example, animal-based diets decrease the levels of *Firmicutes*, which metabolize plant polysaccharides, while increasing the abundance of bile-tolerant microorganisms (34). The efficacy of no-drug therapy is restricted by the lifestyle of T2DM patients. Therefore, more gut microbiota-targeting drugs are worth exploring. The safety of SAL has been widely verified in clinical trials (35, 36). The function of SAL in regulating glucose and iron metabolism by affecting the gut microbiota in the present study indicated that SAL could be effective in treating T2DM along with already known treatments.

In conclusion, the present study showed that SAL could reduce glucose levels and protect against diabetic cardiomyopathy in diabetic mice. The therapeutic effect of SAL on diabetes might be mediated by changes in the gut microbiota the regulation of iron metabolism. The findings suggested that SAL was a promising complementary option for diabetes. The efficacy and safety of SAL are worth investigating in the clinic in the future.

## Data availability statement

The datasets presented in this study can be found in online repositories. The names of the repository/repositories and accession number(s) can be found below: https://www.ncbi.nlm.nih.gov/, PRJNA868147.

## Ethics statement

The animal study was reviewed and approved by the ethics committee of Hospital of Chengdu Office of People’s Government of Tibetan Autonomous Region.

## Author contributions

JShi designed the experiments and prepared the manuscript. QZ helped with experiments. DH performed the data analysis. HM designed the detection index. SW collected mouse faeces. CZ, SYW and SC helped with sample storage and extraction. TF and JSha revised the manuscript. All authors contributed to the article and approved the submitted version.

## Funding

This work was supported by the Natural Science Foundation of Tibet Autonomous Region (XZ202101ZR0101G); the Key Research Projects of the Hospital of Chengdu Office of People’s Government of Tibetan Autonomous Region (Hospital.C.T.) (QH-1(2019)-03); and the Research Project of Sichuan Medical Association (S19072).

## Acknowledgments

The authors are grateful to all members of the laboratory for their continuous technical advice and helpful discussion.

## Conflict of interest

The authors declare that the research was conducted in the absence of any commercial or financial relationships that could be construed as a potential conflict of interest.

## Publisher’s note

All claims expressed in this article are solely those of the authors and do not necessarily represent those of their affiliated organizations, or those of the publisher, the editors and the reviewers. Any product that may be evaluated in this article, or claim that may be made by its manufacturer, is not guaranteed or endorsed by the publisher.
